# Effectiveness of Oral Health Education and Interventions in Improving Oral Health Outcomes in Type II Diabetes Mellitus Patients: A Prospective Study

**DOI:** 10.7759/cureus.58227

**Published:** 2024-04-14

**Authors:** Sowmya S, Sangavi R

**Affiliations:** 1 Oral Medicine, Radiology and Special Care Dentistry, Saveetha Dental College and Hospitals, Saveetha Institute of Medical and Technical Sciences, Chennai, IND

**Keywords:** generalised periodontitis, oral health, oral manifestation, blood glucose level, diabetic mellitus

## Abstract

Introduction

Diabetes mellitus (DM) is a group of metabolic diseases characterized by hyperglycemia resulting from defects in insulin secretion, insulin action, or both. The study aimed to evaluate the effectiveness of oral health education and intervention in improving oral health outcomes in type 2 DM (T2DM) patients.

Methods

The present study was conducted in the Department of Oral Medicine and Radiology between February 2023 and August 2023 at Saveetha Dental College and Hospitals in Chennai, India. All of the patients in the study had T2DM with oral manifestations. This study enrolled 105 participants, of whom 63 were female and 42 were male. A standard pro forma was given to all the participants, and the findings were recorded. The pro forma comprises different oral manifestations, blood glucose levels, the Decayed, Missing, and Filled Teeth (DMFT) index, and Russell’s periodontal index. The results were then statistically analyzed.

Results

This study of 105 individuals with T2DM (60% females and 40% males) revealed significant oral health challenges: 33% had periodontitis, 20% had gingivitis, 5% had lichenoid reactions, 23% had xerostomia, 11% had halitosis, and 8% had candidiasis, illustrating diabetes’ impact on dental health. Following appropriate, tailor-made treatment for individual patients, such as scaling, root planning, oral hygiene education, pharmacotherapy, and post-intervention, the prevalence of complications notably decreased by 61%. A total of 7% of patients had gingivitis, 11% had periodontitis, 12% had xerostomia, 4% had halitosis, 2% had candidiasis, and 1% had lichenoid reactions, respectively. This highlights the importance of regular oral care positively impacting diabetes patients, with 61% experiencing improved oral health and 39% experiencing no improvement.

Conclusion

This study provides compelling evidence for the effectiveness of oral health education and interventions in improving oral health outcomes in T2DM patients. This approach offers a promising strategy for managing the oral health complications associated with diabetes and improving this population’s overall health and well-being.

## Introduction

Diabetes mellitus (DM) is a long-term metabolic disease characterized by high blood glucose levels followed by reduced insulin production in peripheral tissues [1]. An estimated 463 million people worldwide, or 9.3% of the total population, had DM in 2019. By 2045, this percentage is expected to increase dramatically to 10.9% (or 700 million people) [2]. DM is a widespread disease affecting nearly every organ in the body. It has various consequences in the human body, such as diabetic retinopathy, diabetic neuropathy, peripheral artery diseases, and diabetic nephropathy [3]. Type 2 DM (T2DM) affects adults; however, type 1 diabetes is more frequent in children and adolescents. Individuals diagnosed with T2DM typically exhibit insulin resistance, which alters the target cells’ utilization of endogenously produced insulin [4]. In addition, T2DM causes several problems with oral wellness, primarily periodontal disorders [5-8]. Research has consistently highlighted the link between uncontrolled diabetes and a heightened risk of various oral health issues. Notably, individuals with poorly managed diabetes are more susceptible to recurrent bacterial and fungal infections. Various studies have shown an association of diabetes with a burning mouth syndrome, dysgeusia, sialadenosis, candidiasis, lichen planus, hyposalivation, or xerostomia [9,10]. Several studies have shown that diabetic individuals have a lack of awareness of fundamental oral health problems. In addition, the majority of these studies demonstrated that relatively few patients with diabetes routinely visit a dentist for dental examinations, and many patients were unaware of the impact of diabetes on oral health [11-13]. Periodic dental checkups provide chances for adult dental disease prevention, detection, and treatment. Furthermore, regular dental care could enhance glucose control in those with poorly controlled diabetes [14].

Oral physicians are important in managing the oral health of patients with T2DM. They are trained to identify signs and symptoms of T2DM in the oral cavity, including oral candidiasis (thrush), gingivitis and periodontitis, xerostomia, burning mouth syndrome, delayed wound healing, and oral lichenoid reactions (OLRs). In addition, oral physicians can collaborate with primary care physicians to identify patients who might be at risk of developing T2DM based on oral findings. Dental investigations, oral prophylaxis, and proper dental care may decrease the incidence of oral diseases [15,16]. The present study combines oral health education with a dental intervention. This comprehensive approach allows for assessing the combined effect of both oral hygiene education and dental intervention strategies on improving oral health outcomes. The present prospective study was conducted to determine the effectiveness of oral health education and interventions in improving oral health outcomes in T2DM patients.

## Materials and methods

A prospective study was conducted in the Department of Oral Medicine and Radiology from February 2023 to August 2023 at Saveetha Dental College and Hospitals in Chennai, India. The study was approved by the Saveetha Dental College Institutional Human Ethical Committee (approval number: IHEC/SDC/OMED-2202/23/211). Before beginning the study, each patient was given comprehensive information about the study and had to provide written informed consent before participating. This study investigated the oral health of 105 individuals diagnosed with T2DM.

Patient selection

Before the study, each patient received detailed information and provided written informed consent. Blood tests were then conducted to assess fasting blood glucose, postprandial blood glucose, and glycosylated hemoglobin (HbA1c) levels. Patients with fasting glucose levels exceeding 110 mg/dL, postprandial levels exceeding 200 mg/dL, and HbA1c levels greater than 7 were included in the study. Additionally, complete medical histories were obtained. Patients not already under diabetic care were referred to general physicians for management.

Training and calibration

Two researchers underwent a two-month training and calibration process to select patients for this study. The training included reviewing eligibility criteria and carefully assessing patients for inclusion. Calibration involved the researchers practicing patient selection under the supervision of more experienced researchers. Interexaminer reliability, measured using kappa statistics, was found to be 0.86.

Sample size calculation

In the present study, a simple randomized sampling method was employed. The sample size for this study was determined based on earlier research on a related subject using G*Power, a statistical software commonly used for sample size calculation in research studies. This approach ensured that the sample size was adequate to achieve the study’s objectives and maintain statistical power.

Inclusion criteria

This study enrolled participants with a diagnosis of T2DM and under treatment, patients aged greater than 18 years, both males and females, and patients from the south Chennai zone.

Exclusion criteria

Patients with a history of any malignancies undergoing chemotherapy or radiotherapy that significantly impact oral health were excluded from the present study. This exclusion was necessary to avoid potential interference with the study’s results. Additionally, other comorbidities such as hypertension, epilepsy, coronary artery diseases, etc. were considered in the exclusion criteria.

Oral health assessment and interventions

The assessment included a complete oral screening, calculation of the patient’s Decayed, Missing, and Filled Teeth (DMFT) score and Russell’s periodontal score, and examination of various oral manifestations. Each participant underwent a comprehensive oral examination. Common oral manifestations observed included periodontitis, gingivitis, tooth mobility, tooth loss, dental caries, and halitosis. To address these issues, various interventions were implemented:

Oral Prophylaxis

Scaling and root planning procedures were performed.

Dental Restorations

Tooth decay was treated with appropriate restorations.

Targeted Medications

Specific conditions received targeted medications, such as the following:

Lichenoid reactions: Old metallic restorations were removed and replaced with composite restorations, which were prescribed with 0.1% triamcinolone acetonide applied thrice daily for two weeks.

Candidiasis: A topical antifungal agent (1% clotrimazole) was applied thrice daily for two weeks.

Halitosis: Chlorhexidine mouthwash (15 mL, twice daily) is used for rinsing and expectoration after brushing, following oral prophylaxis.

Xerostomia: Patients with xerostomia were advised to take a chewable vitamin C tablet (500 mg) thrice daily for two weeks.

Oral hygiene instruction: Personalized instructions were provided to improve daily brushing and flossing techniques.

Follow-up

The importance of routine dental checkups was emphasized. Patients were initially scheduled for bimonthly (every two months) follow-ups, with reviews at three-month and six-month intervals thereafter. This regular monitoring played a significant role in maintaining their improved oral health.

Statistical analysis

The information was imported into Microsoft Excel 2016 (Microsoft Corporation, Redmond, Washington, United States) and then subjected to analysis using IBM SPSS Statistics for Windows, Version 29.0 (Released 2022; IBM Corp., Armonk, New York, United States). Descriptive statistics were utilized to explain the data using frequency analysis and percentage analysis, while mean and standard deviation were employed to characterize the continuous variables. Additionally, a repeated measures ANOVA was performed.

## Results

**Figure 1:**
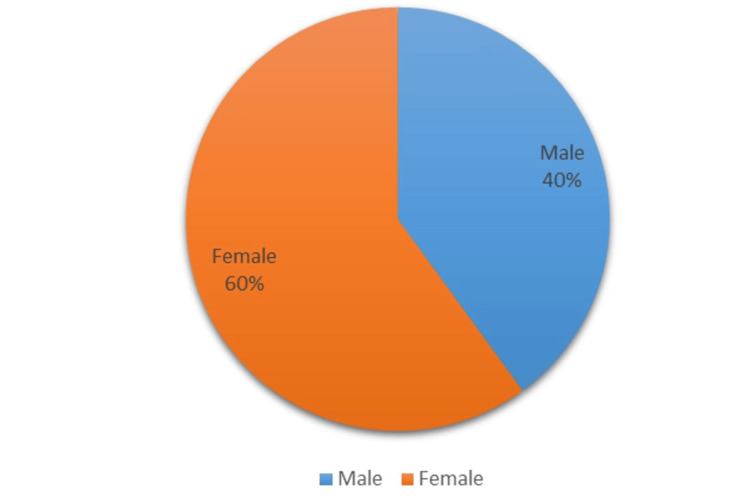
Gender distribution of T2DM patients T2DM, type 2 diabetes mellitus

**Figure 2:**
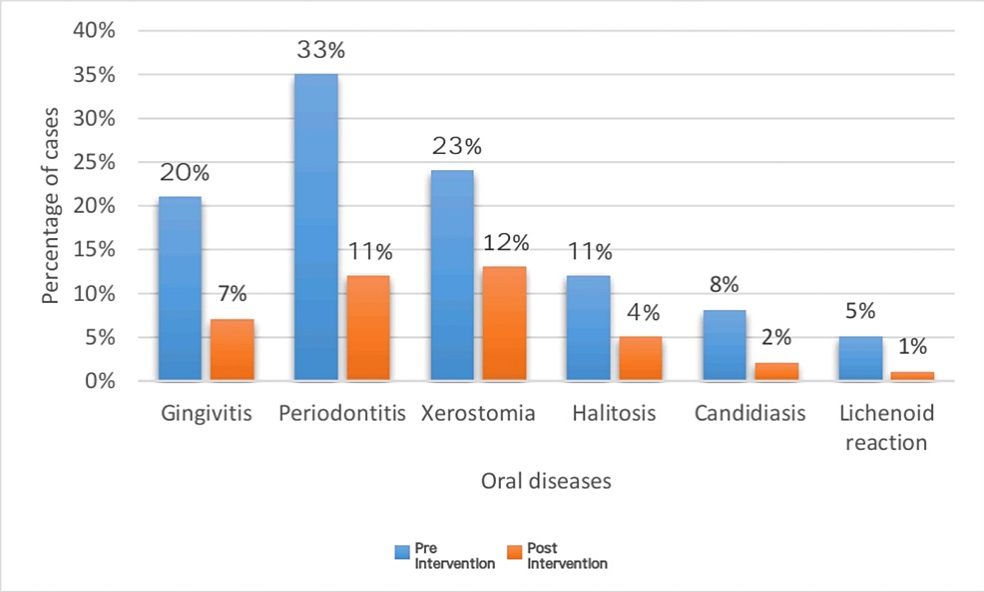
Pre- and post-intervention of oral diseases in diabetic patients

**Figure 3:**
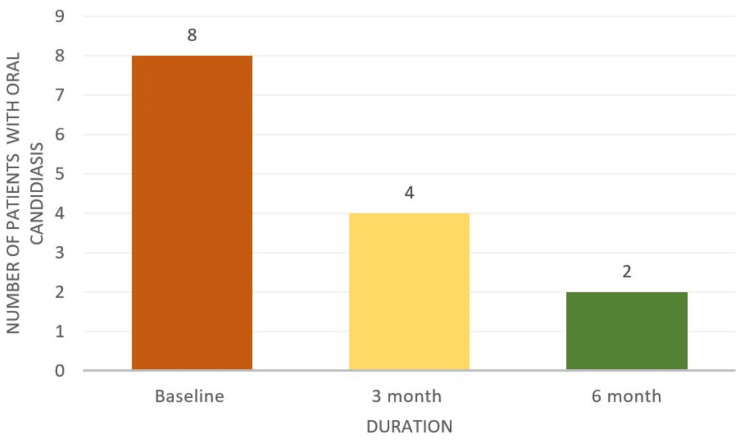
Effect of intervention on candidiasis

**Figure 4:**
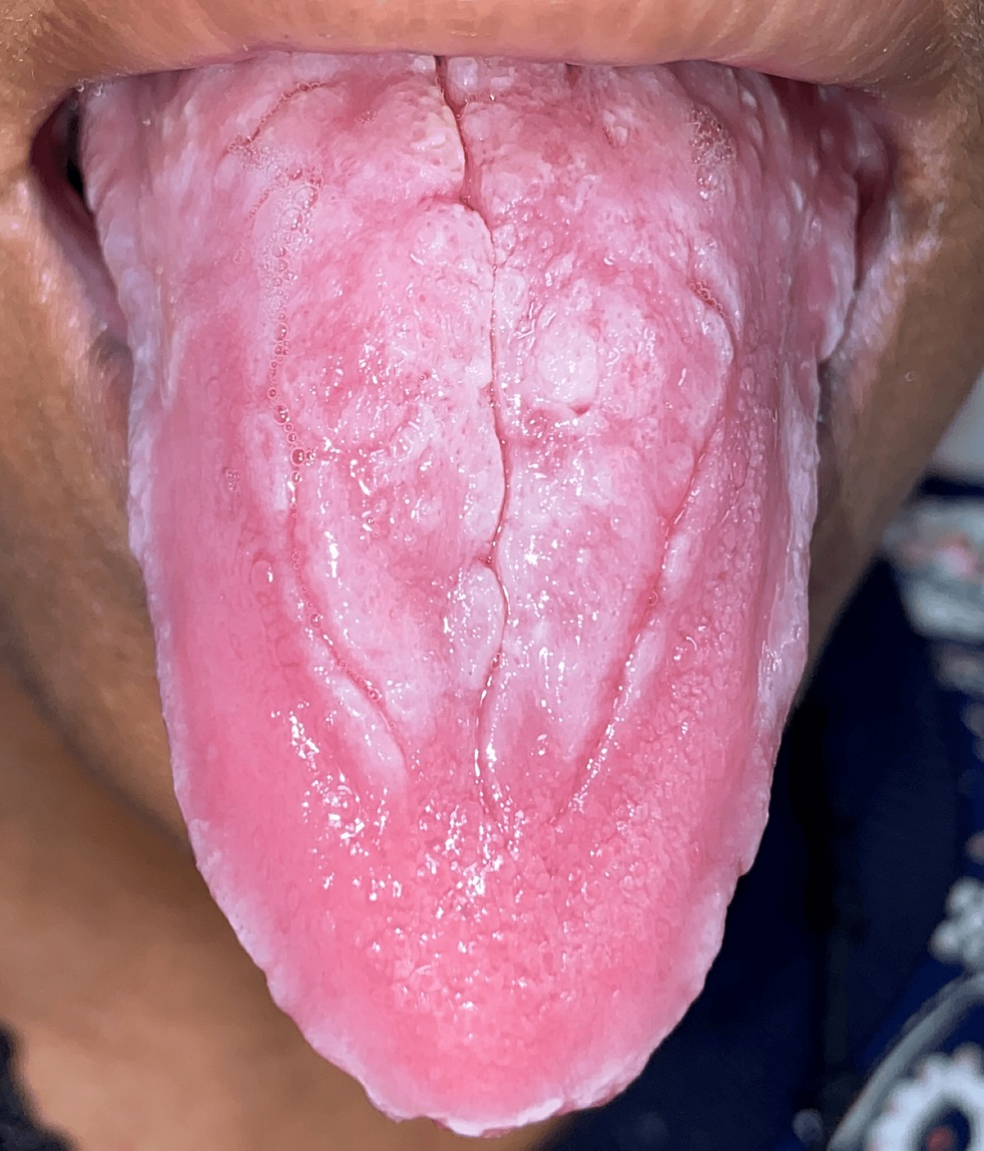
Candidiasis on the dorsal surface of the tongue

**Figure 5:**
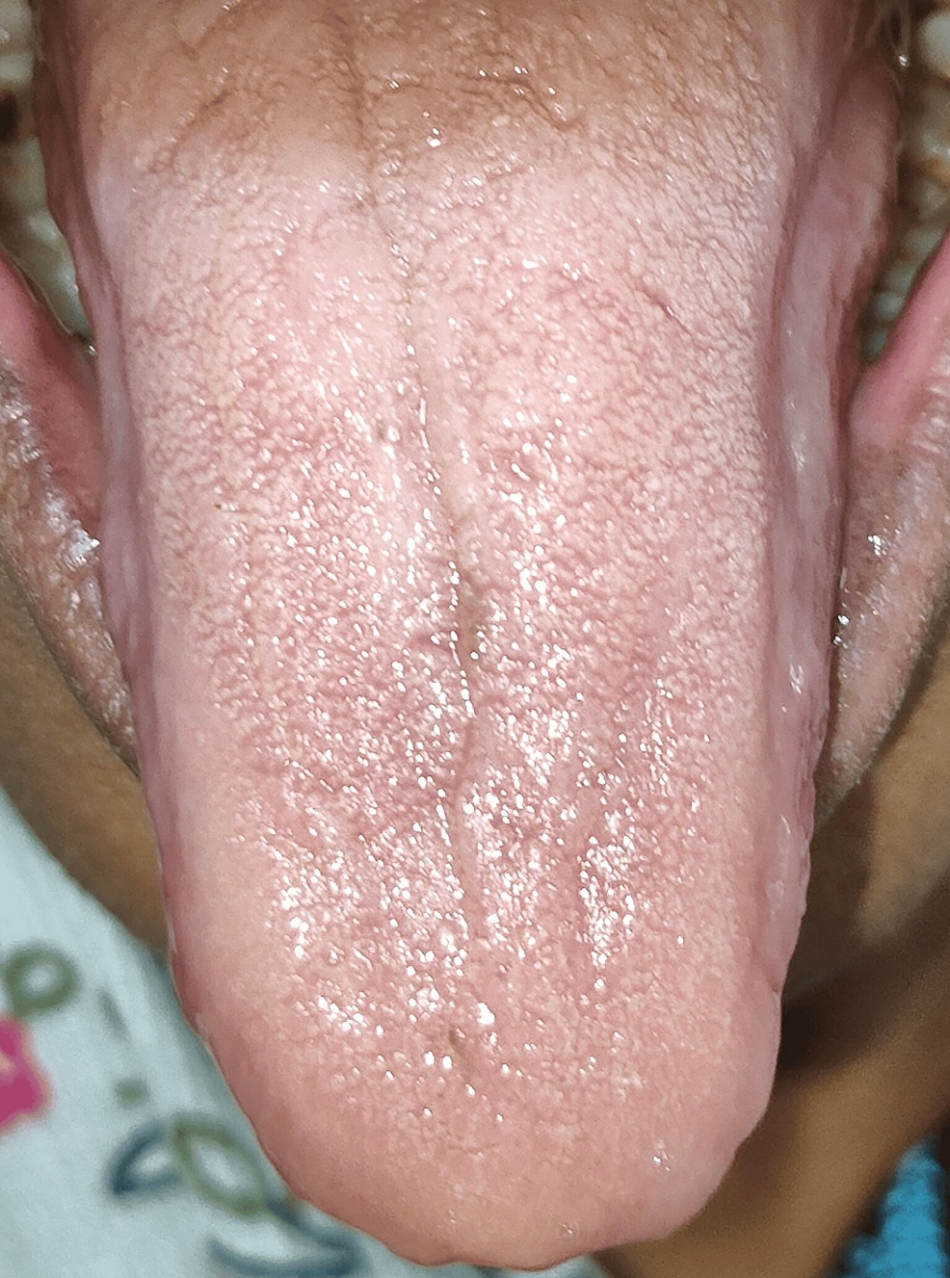
Post-intervention reveals the healing of candidiasis

**Figure 6:**
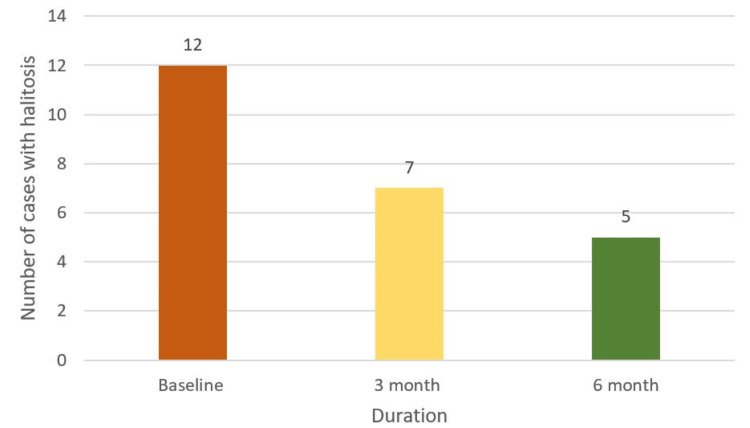
Effect of intervention on halitosis

**Figure 7:**
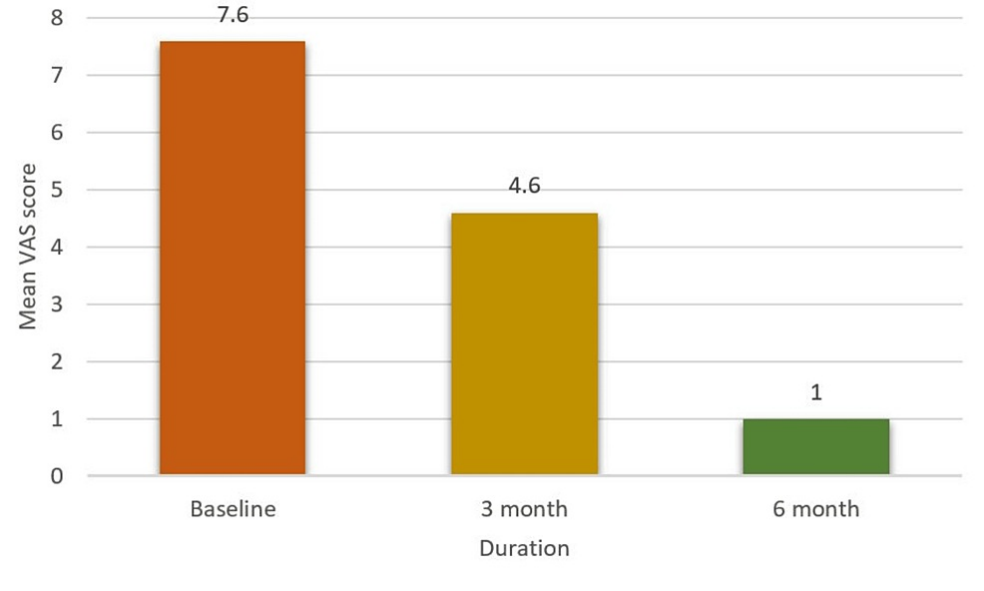
Effect of intervention on lichenoid reaction VAS, Visual Analogue Scale

**Figure 8:**
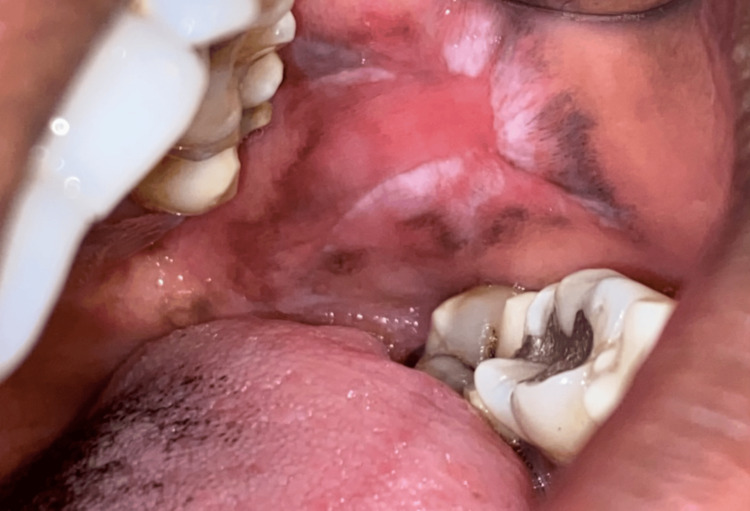
Lichenoid reaction seen in the left buccal mucosa

**Figure 9:**
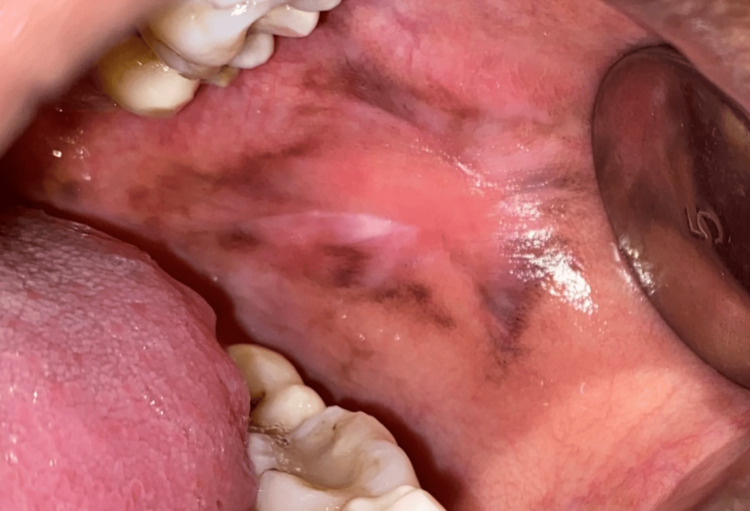
Post-intervention reveals healing of lesion

This study aims to assess the effectiveness of oral health education and interventions in improving oral health outcomes in T2DM patients. Among 105 participants (60% females and 40% males) (Figure 1), a significant portion experienced challenges: 33% had periodontitis, 20% had gingivitis, 5% suffered from lichenoid reactions, 23% suffered from xerostomia, and 11% and 8% experienced halitosis and candidiasis, respectively (Figure 2). These results highlight the significant effect of DM on dental health. Further analysis revealed the effectiveness of timely interventions. After receiving dental treatments like scaling and root planning, along with instruction on oral hygiene practices and pharmacotherapy for oral mucosal lesions, overall oral health showed a remarkable improvement. The intervention had a positive impact on candidiasis, with eight patients having candidiasis at baseline. During treatment, this number gradually decreased to four, and with regular follow-up and intervention, it further reduced to two within six months (Figure 3). It revealed candidiasis on the dorsum surface of the tongue (Figure 4), which healed post-pharmacological intervention (Figure 5). The intervention also showed a positive impact on halitosis. Initially, 12 patients had halitosis, which decreased to seven during treatment and was further reduced to five with regular follow-up within six months (Figure 6). In individuals with lichenoid reactions, the intervention consistently decreased the mean Visual Analogue Scale (VAS) score over time. The VAS score decreased from 7.6 at baseline to 4.6 at three months and to 1 at six months (Figure 7). It reveals a case of lichenoid reaction on the left buccal mucosa (Figure 8) that healed post-intervention (Figure 9).

**Figure 10:**
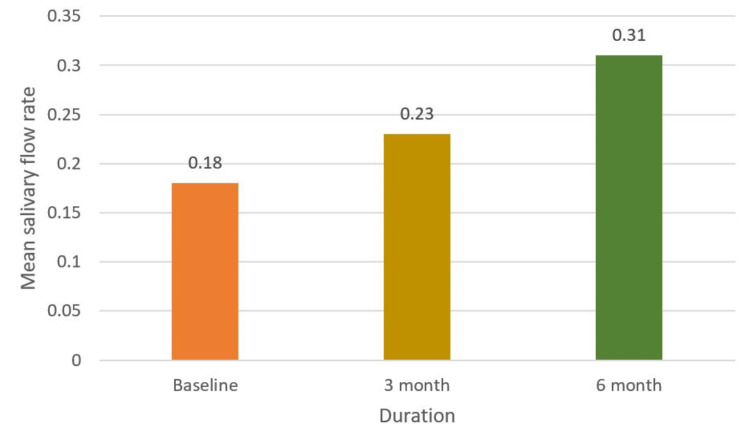
Effect of intervention on xerostomia

**Figure 11:**
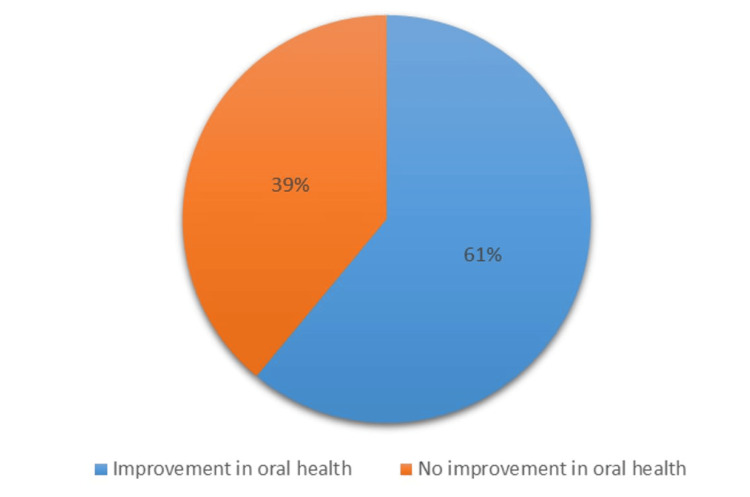
Pre- and post-intervention improvements in oral health

Similarly, the intervention positively affected the mean salivary flow rate in individuals with xerostomia, showing a steady increase over time. The mean flow rate increased from 0.18 mL/min at baseline to 0.23 mL/min at three months and further improved to 0.31 mL/min at six months (Figure 10). The mean DMFT score and mean Russell’s score at baseline, third month, and sixth month, along with their corresponding F values, indicate the significance of changes over time. The mean DMFT score decreased progressively from baseline to the third month and further by the sixth month, reflecting an enhancement in oral health concerning decayed, missing, and filled teeth throughout the study duration. Similarly, the mean Russell’s score exhibited a decline from baseline to the third and sixth months, indicating an improvement in oral hygiene status over the study period. The substantial F values imply significant differences in the mean DMFT score and Russell’s score across the three time points, indicating that the changes in these scores over time are unlikely due to random chance and are more likely due to the intervention or treatment being studied (Table 1). The prevalence of various complications decreased significantly. On post-intervention, only 7% of participants still had gingivitis, 11% had periodontitis, 12% experienced xerostomia, 4% had halitosis, a mere 2% still suffered from candidiasis, and only 1% suffered from lichenoid reaction (Figure 2). The post-intervention oral health improved by 61%, and 39% of patients showed no improvement (Figure 11).

**Table 1:** Mean DMFT score and mean Russell’s score for baseline, third month, and sixth month DMFT, Decayed, Missing, and Filled Teeth

	Mean DMFT score	Mean Russell’s score
Baseline	2.15 ± 0.52	1.36 ± 0.25
Third month	1.44 ± 0.46	0.86 ± 0.30
Sixth month	0.91 ± 0.40	0.78 ± 0.31
F value	275.518	190.801
P value	0.001	0.001

## Discussion

The objective of the study is to assess the effectiveness of oral health education and interventions in improving oral health outcomes in T2DM. Periodontal diseases, such as gingivitis and periodontitis, are the most common oral complications associated with diabetes [1]. Additionally, other oral health conditions like xerostomia with burning mouth syndrome, dysgeusia, sialadenosis, fungal infections (e.g., candidiasis), and less frequent lesions like lichenoid reactions have been reported in diabetic individuals [13]. T2DM patients tend to have poor oral health compared to healthy individuals, with an increased risk of compromised periodontal status and high DMFT scores [14].

The study conducted by Ciarambino et al. in the year 2022 shows that the gender distribution of T2DM prevalence is noteworthy. While globally, men have a higher prevalence of diabetes, women diagnosed with T2DM outnumber men [12]. The present study found that, out of 105 cases, 40% were male and 60% were female. Our result is consistent with the result derived by Ciarambino et al. Females were mostly affected by T2DM because certain hormonal imbalances during menstruation, pregnancy, and menopause could affect insulin sensitivity and metabolism. It is important to remember that T2DM is a complex condition with multiple contributing factors. Research suggests that differences in body fat distribution, with women often having more abdominal fat (visceral fat) linked to insulin resistance, may also play a role [17,18].

Several studies have confirmed the strong association between periodontal disease and diabetes. In a 2019 study conducted by Rohani, a significantly higher prevalence of severe periodontitis was observed in diabetic patients (59.6%) compared to nondiabetics (39%) [19]. The present study corroborates these findings, with an even higher prevalence of periodontitis (33%) and substantial tooth loss, potentially linked to this increased susceptibility to periodontal disease. Patients with diabetes are more likely to develop periodontal disease due to irregularities in collagen metabolism, reduced polymorphonuclear leucocyte activity, and the development of a glycosylated end product that compromises the stability of collagen and vascular integrity. The final glycosylated products elevate interleukin-1 and tumor necrosis factor alpha and bind to the receptors on macrophages and monocytes, increasing the sensitivity to tissue damage [20,21].

The study conducted by Al-Zahrani et al. in 2011 suggests a positive relationship between HbA1c levels and halitosis among diabetic patients. The present studies show that 11% of diabetic patients are associated with halitosis, and 23% of patients are diagnosed with xerostomia. Diabetes can damage the cholinergic parasympathetic nerves that control the salivary glands, leading to a condition called diabetic autonomic neuropathy. This neuropathy can impair the ability of the glands to receive signals from the brain, ultimately reducing saliva production, which leads to a condition called xerostomia. Saliva plays a critical role in cleansing the oral cavity by removing debris and bacteria that can contribute to halitosis. Without adequate saliva, these bacteria, such as *Bacteroides* spp., *Actinomyces* spp., *Eubacterium *spp., *Dialister *spp., *Leptotrichia *spp., *Fusobacterium* spp., *Peptostreptococcus *spp., *Prevotella *spp., *Porphyromonas* spp., *Solobacterium *spp., *Selenomonas *spp., *Tannerella forsythia, *and *Veillonella *spp. can thrive and multiply, releasing volatile sulfur compounds responsible for the characteristic bad breath odor [22,23].

There is a well-established link between diabetes and candidiasis. This is due to elevated glucose levels in diabetic individuals, which provide an ideal environment for the growth of* Candida albicans*, significantly increasing the risk of infection. Additionally, poorly managed diabetes weakens the immune system, further reducing the body’s ability to combat fungal infections. A study by Mohammadi et al. in 2016 reported a high prevalence of* Candida *colonization in the oral mucosa of diabetic patients, with 55% carrying *Candida* species. The present study suggests only 8% of cases are associated with candidiasis. The present study is consistent with the previous study [24]. Medication for DM patients, especially several hypoglycemic agents such as glimepiride, could trigger an allergic response that results in a drug-induced lichenoid reaction [25]. According to Oivio et al., in 2020, the prevalence of OLR was 3.5% in the middle-aged northern Finland population [26]. The present study shows that 5% of diabetic patients had a lichenoid reaction. The present study is inconsistent with the previous study; this is due to the diversity of the population and associated systemic conditions. Certain hypoglycemia medications may cause an immunological reaction that results in inflammation and damage to the oral mucosa because of oxidative stress, drug hypersensitivity reactions, and pH changes in the oral cavity [27]. This study investigated the oral health of 105 individuals with T2DM. Notably, they presented with oral diseases like periodontitis and gingivitis, leading to problems like tooth mobility, tooth loss, dental caries, and halitosis.

To address these issues, the patients underwent various interventions, including oral prophylaxis such as scaling and root planning, dental restorations for cavities, and targeted medication for specific conditions such as topical antifungal agent 1% clotrimazole (two to three times a day for two weeks) for candidiasis and treatment for halitosis, which includes chlorhexidine mouthwash. Patients were instructed to use 15 mL (one capful) of undiluted oral rinse, swishing, and then expectorating after brushing, repeating twice a day, in the morning and evening. Halitosis therapy should start immediately after dental prophylaxis is completed. In xerostomia, the patient was advised to take a vitamin C chewable tablet of 500 mg thrice daily for two weeks. Furthermore, they received personalized oral hygiene instructions aimed at improving their daily practices. Importantly, routine dental checkups were emphasized. The patients’ oral health showed significant improvement compared to nondiabetic individuals, with bimonthly recalls playing a crucial role in their progress. In addition, oral physicians can educate patients about how T2DM affects their oral health and the importance of maintaining good oral hygiene practices. Lifestyle factors like diet, smoking cessation, and stress management can significantly impact T2DM and oral health [28].

Overall, post-intervention oral health improved by 61%. Oral physicians play a vital role in managing the oral health of patients with T2DM by providing comprehensive diagnosis, education, intervention, and follow-up care. By working closely with patients and general physicians, oral physicians can help ensure that individuals with T2DM maintain good oral health, which contributes to their overall well-being and quality of life. Regular dental checkups once every six months and consistent oral care are essential for individuals with T2DM. Addressing oral health concerns promptly can prevent their progression and avert serious complications.

Strength

The study’s strength lies in its rigorous design, comprehensive intervention approach, and statistically significant findings, providing valuable insights into improving oral health outcomes in diabetes patients.

Limitation

The study’s limitations include its single-center design with a small sample size, which may restrict the generalizability of the findings to other populations with different demographic and clinical profiles. Conducting prospective studies with larger sample sizes and longer follow-up periods would offer more comprehensive and reliable evidence regarding oral health outcomes in individuals with T2DM.

Future scope

Future research should include longitudinal and multicenter studies with larger sample sizes to understand the long-term impact of oral health interventions on individuals with T2DM. Interventional studies can evaluate the effectiveness of specific oral health interventions, while research on behavioral interventions like diet modifications and smoking cessation can offer insights into holistic diabetes management. Integrating technology, such as telehealth and digital platforms, in delivering oral health education to diabetic patients could improve accessibility and effectiveness. Lastly, conducting health economic evaluations to assess the cost-effectiveness of oral health interventions in diabetes care is crucial for healthcare decision-making.

## Conclusions

DM presents a significant risk factor for various oral health complications, including periodontal diseases, xerostomia, fungal infections, and lichenoid reactions. The present study, conducted among a South Indian population, highlights the effectiveness of oral health education and interventions in improving oral health outcomes for T2DM. Notably, the findings of the present study align with previous research regarding the higher prevalence of periodontal diseases in diabetic patients and the impact of gender on diabetes prevalence. The study underscores the importance of regular dental checkups, personalized oral hygiene instructions, and targeted interventions for managing oral health in individuals with T2DM. The significant improvement in post-intervention oral health, with a 61% improvement rate, emphasizes the crucial role of oral physicians in providing comprehensive care for diabetic patients. Collaboration between oral physicians and general physicians is essential for ensuring that individuals with T2DM maintain good oral health, contributing to their overall well-being and quality of life.

## Figures and Tables

**Figure 12:**
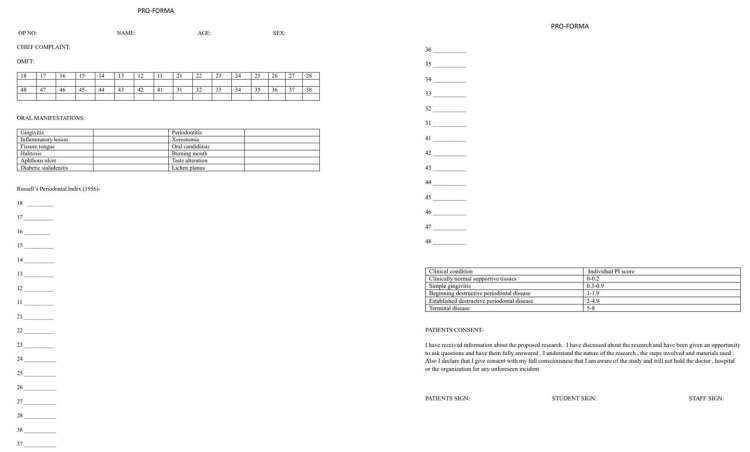
Pro forma for the study
